# Development and validation of risk prediction models for cardiovascular mortality in Chinese people initialising peritoneal dialysis: a cohort study

**DOI:** 10.1038/s41598-018-20160-3

**Published:** 2018-01-31

**Authors:** Dahai Yu, Yamei Cai, Ying Chen, Tao Chen, Rui Qin, Zhanzheng Zhao, David Simmons

**Affiliations:** 1 Department of Nephrology, the First Affiliated Hospital, Zhengzhou University, Zhengzhou, 450052 China; 2 Arthritis Research UK Primary Care Centre, Research Institute for Primary Care & Health Sciences, Keele University, Keele, ST5 5BG UK; 3 Tropical Clinical Trials Unit, Department of Clinical Sciences, Liverpool School of Tropical Medicine, Pembroke Place, Liverpool, L3 5QA UK; 4 Jiangsu Province Hospital on Integration of Chinese and Western Medicine, Nanjing, 210028 China; 5 Western Sydney University, Campbelltown, Sydney, NSW 2751 Australia

## Abstract

Cardiovascular disease is the leading cause of death among patients receiving peritoneal dialysis. We aimed to develop and validate a risk prediction model for cardiovascular death within 2 years after the initiation of peritoneal dialysis (PD). A cohort including all patients registered with the Henan Peritoneal Dialysis Registry (HPDR) between 2007 and 2014. Multivariate logistic regression analysis was used to develop the risk prediction model. The HPDR data was randomly divided into two cohorts with 60% (1,835 patients) for model derivation, and 40% (1,219 patients) for model validation. The absolute rate of cardiovascular mortality was 14.2% and 14.4 in the derivation and validation cohort, respectively. Age, body mass index, blood pressure, serum lipids, fasting glucose, sodium, albumin, total protein, and phosphorus were the strongest predictors of cardiovascular mortality in the final model. Discrimination of the model was similar in both cohorts, with a C statistic above 0.70, with good calibration of observed and predicted risks. The new prediction model that has been developed and validated with clinical measurements that are available at the point of initiation of PD and could serve as a tool to screen for patients at high risk of cardiovascular death and tailor more intensive cardio-protective care.

## Introduction

In developing countries, the risk of mortality with end stage renal disease (ESRD) remains high, with cardiovascular disease (CVD) the primary cause of death^1,2^. Technological advances have allowed peritoneal dialysis (PD) to be increasingly applied to ESRD patients, especially in developing countries^3–5^. Despite these advances, mortality risk often remains high among ESRD patients accepting PD care^2^. Several risk prediction models have been developed to predict the future all-cause mortality risk among patients undergoing hemodialysis and PD, in both developed countries and developing coun tries^5–8^.

However, although CVD is the primary cause of mortality among patients with ESRD, for which successful preventative interventions exist, no risk prediction models have been developed identify patients receiving PD care, who have not received appropriate interventions previously and could benefit from cardio-protective interventions, or who have received interventions and not attained control threshold and could benefit from intensive cardio-protective interventions. Moreover, unlike patients under health care systems in many western countries, in developing countries, valid health information is difficult to obtain as a result of limited primary or secondary care systems able to accurately report pre-existing conditions and comorbidities^4,9,10^. Depending upon patient self-reported history may be misleading in this setting, we therefore wished to develop risk equations that could predict future CVD mortality largely using objective clinical data likely to be available in developing countries. Our study aimed to develop and validate a risk prediction model for predicting 2-year CVD mortality among people initialising PD care.

## Results

### Study participants

In our derivation cohort, we analysed information on 1,835 patients with 261 cardiovascular deaths within 2 years of initialisation of PD. The validation cohort had information on 1,219 patients with 176 cardiovascular deaths. Table 1 summarises the basic characteristics of the study population: patients in both cohorts had broadly similar characteristics.

**Table 1:** Baseline Characteristics of study populations.

Candidate Predictors	Derivation	Validation
N	1835	1219
Cardiovascular Deaths, n (%)	261 (14.2)	176 (14.4)
Male Gender, n (%)	1074 (58.5)	716 (58.2)
Primary Glomerular Disease, n (%)	871 (41.3)	575 (42.0)
Age, years	48.8 (38.0 to 59.0)	49.0 (38.5 to 59.0)
Haemoglobin, g/L	88.0 (75.0 to 102.0)	88.0 (74.0 to 102.0)
Packed cell volume	19.4 (2.3 to 27.8)	18.5 (2.8 to 27.2)
Reticulocyte, %	35.0 (13.2 to 60.2)	34.0 (12.4 to 61.3)
Phosphorus, mg/dl	1.8 (1.4 to 2.2)	1.8 (1.4 to 2.2)
Albumin, g/L	33.5 (29.8 to 37.9)	33.7 (29.6 to 37.7)
Total iron binding capacity, μmol/L	45.0 (34.9 to 52.0)	44.5 (35.0 to 53.0)
FeTIBC, mmol/L	24.7 (21.0 to 41.61)	25.4 (20.8 to 40.4)
Creatinine, µmol/L	842.6 (634.0 to 1061.0)	822.5 (631.0 to 1071.0)
estimated Glomerular Filtration rate, mL/min/1.73 m^2^	4.8 (3.6 to 6.9)	4.7 (3.5 to 6.5)
Transferrin, mg/dl	200.0 (125.0 to 429.7)	822.5 (631 to 1071)
Total protein, g/L	57.8 (52.3 to 63.1)	57.5 (51.8 to 63.0)
Prealbumin, mg/L	291.0 (199.3 to 362.0)	298.0 (188.0 to 364.0)
Total Cholesterol, mmol/L	4.4 (3.6 to 5.2)	4.5 (3.6 to 5.2)
Low density lipoprotein, mmol/L	2.6 (1.9 to 3.4)	2.6 (2.0 to 3.4)
Fasting glucose, mmol/L	5.0 (4.3 to 6.0)	4.9 (4.3 to 5.9)
Sodium, mEq/L	140.0 (136.9 to 142.5)	139.7 (136.8 to 142.0)
C-reaction protein, mg/dl	2.6 (1.0 to 5.7)	2.1 (1.0 to 4.9)
Body mass index, kg/m^2^	22.7 (20.6 to 24.9)	22.7 (20.8 to 24.8)
Systolic blood pressure, mmHg	145.0 (135.0 to 159.0)	143.0 (135.0 to 158.2)
Diastolic blood pressure, mmHg	86.0 (80.0 to 95.0)	87.0 (80.0 to 95.0)
Cardiovascular diseases, n (%)	842 (45.9)	542 (44.5)
Type 2 Diabetes, n (%)	261 (14.2)	188 (15.4)
Taking antihypertensive treatment, n (%)	765 (41.7)	492 (40.4)

Binary variable are displayed as numbers (percentage) and continuous variables are displayed as median (interquartile).

### Model development, performance measure, and validation

**Figure 1:**
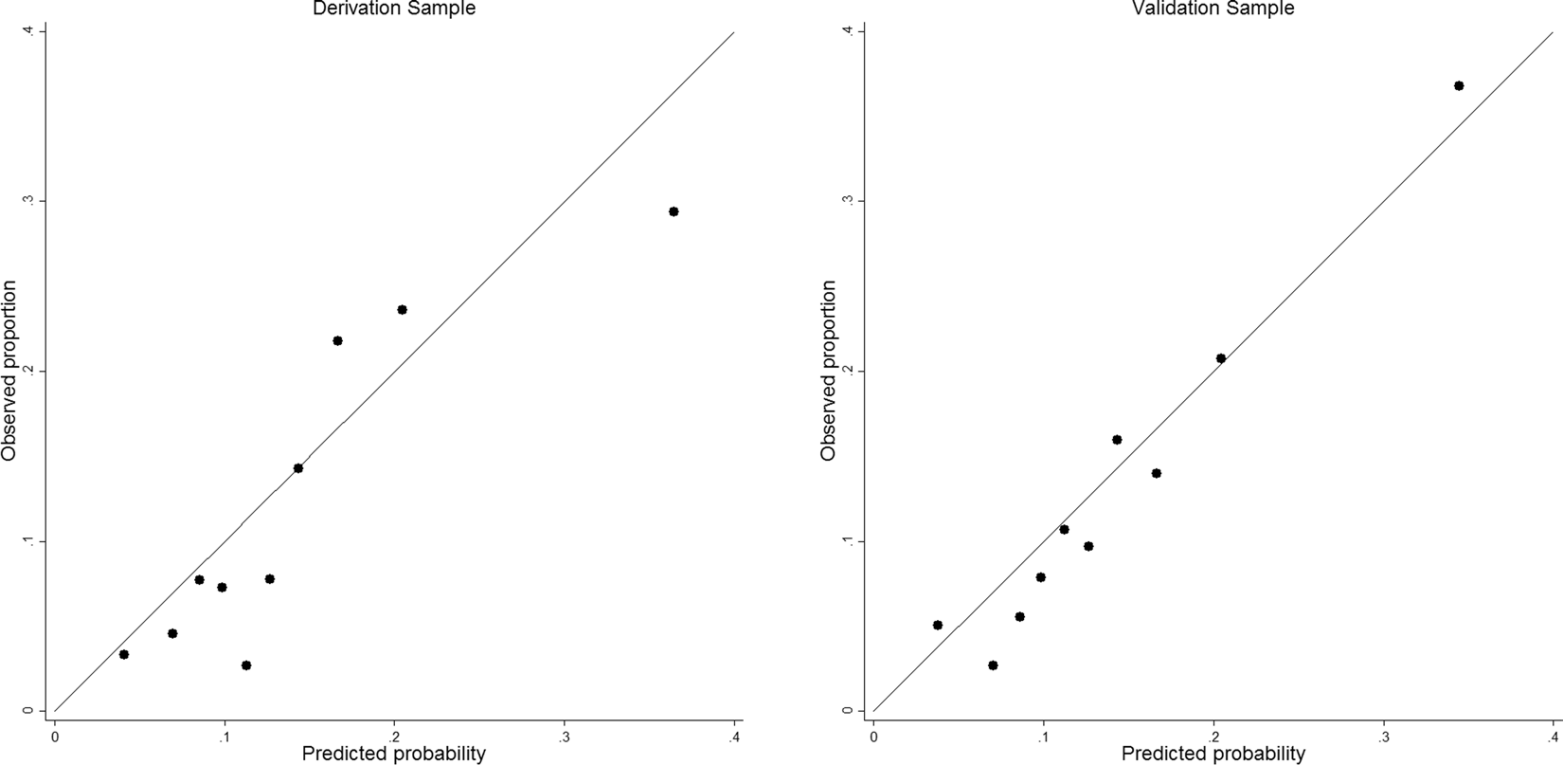
Assessing calibration in the derivation cohort (left) and the derivation cohort (right).

Univariate association between cardiovascular mortality and candidate predictors are listed in supplemental Table 1. Of the 26 candidate predictors, 11 were statistically significantly associated with cardiovascular mortality in our final multivariable model (Table 2). Table 3 shows apparent and internal validation performance statistics of our risk prediction model. After adjustment for optimism, our final risk prediction model was able to discriminate patients receiving PD care with and without cardiovascular mortality with a C statistics of 0.7318 (95% confidence interval 0.6988 to 0.7648). The agreement between the observed and predicted proportion of events showed good apparent calibration (Fig. 1, left), but a uniform shrinkage factor of 0.026 was needed to adjust predictors coefficients in the final model for optimism (Table 3). Box-1 shows our final risk prediction model, including real examples to illustrate the risk prediction equations.

**Table 2:** Final multivariate analysis for cardiovascular mortality risk within two years of initialisation of peritoneal dialysis in derivation cohort.

Predictors	Coefficient	95% Confidence Interval
Age	0.02041	(0.018959 to 0.021856)
(Body mass index/10)^2	−0.5102	(−0.59201 to −0.42838)
(Body mass index/10)^2*ln(Body mass index/10)	0.35498	(0.299653 to 0.410314)
(Phosphorus/10)^−0.5	−0.582	(−0.70562 to −0.45848)
(Phosphorus/10)^3	2.60171	(1.380815 to 3.822601)
(Total Protein/100)^−2	−0.002	(−0.0062 to 0.002175)
(Total Protein/100)^−0.5	−0.6709	(−0.88517 to −0.45664)
[(Total Cholesterol + 1.673428429186277)/10]^0.5	0.91848	(−0.5990 to 2.435950)
[(Total Cholesterol + 1.673428429186277)/10]^0.5*ln[(Total Cholesterol + 1.673428429186277)/10]	−1.3419	(−2.37972 to −0.30414)
(Sodium/100)^3	−1.0792	(−1.43245 to −0.72589)
(Sodium/100)^3*ln(Sodium/100)	1.09368	(0.534857 to 1.652509)
(Systolic blood pressure/100)^−2	−0.2414	(−0.52917 to 0.046443)
(Systolic blood pressure/100)^−2*ln(Systolic blood pressure/100)	−0.6225	(−1.51146 to 0.266407)
(Diastolic blood pressure/100)^−2	−0.4171	(−0.58956 to −0.24465)
(Diastolic blood pressure/100)^−2*ln(Diastolic blood pressure/100)	−0.4521	(−0.64874 to −0.25555)
Albumin	−0.0791	(−0.08283 to −0.07531)
Low density lipoprotein	−0.0679	(−0.08497 to −0.05092)
Fasting glucose	0.01807	(0.011513 to 0.024625)
Constant	4.08926	(2.363196 to 5.815327)

**Table 3:** Model diagnostics (with 95% CI).

Measure	Derivation				Validation
	Apparent performance	Test performance	Average optimism	Optimism corrected	
C statistic	0.7372 (0.7049 to 0.7695)	0.7318 (0.6988 to 0.7648)	+0.010	0.7272 (0.6949 to 0.7595)	0.7205 (0.6798 to 0.7613)
Calibration slope	1.2960 (0.7752 to 1.8169)	1.096 (0.5752 to 1.5569)	+0.260	1.0360 (0.5152 to 1.5569)	1.0276 (0.7332 to 1.3221)

Applying our final risk prediction model (supplemental Box-S1) to the validation cohort gave a C statistic of 0.7205 (0.6798 to 0.7613) and good calibration (Fig. 1, right), with calibration slope only slightly above 1 (Table 3). The performance of our model at various arbitrary thresholds in shown in Table 4.

**Table 4:** Predicted risk of cardiovascular mortality the validation cohort based on various cut-offs.

	Cut-off (%) for risk scores	Mean predicted risk (%)	Sensitivity (%)	Specificity (%)	Positive Predictive Value (%)	Observed risk %
Cardiovascular mortality						
Top 5%	29.12	40.30	18.80 (13.30 to 25.30)	96.00 (94.70 to 97.00)	40.70 (29.90 to 52.20)	40.74
Top 10%	23.32	34.48	28.40 (21.90 to 35.70)	92.80 (91.20 to 94.20)	36.80 (28.70 to 45.50)	36.76
Top 15%	20.43	30.42	36.40 (29.30 to 43.90)	88.60 (86.60 to 90.30)	32.00 (25.60 to 38.90)	32.00
Top 20%	18.31	27.50	44.30 (36.80 to 52.00)	83.80 (81.60 to 85.90)	28.80 (23.50 to 34.60)	28.78
Top 25%	16.78	25.70	51.10 (43.50 to 58.70)	79.90 (77.50 to 82.10)	27.30 (22.50 to 32.40)	27.27

## Disscussion

### Main findings

We have developed and validated a new risk prediction model to calculate the absolute risk of cardiovascular mortality during the first 2 years of initialisation of PD in a representative sample of patients receiving PD care in Henan, the province with the largest population size in China. Overall, our prediction model had good calibration and useful discrimination, with a C statistic of greater than 0.70 in both derivation and calibration cohorts data.

### Strength and limitation of study

Our risk prediction algorithm has several advantages over those in use in many developing countries. The model is based on absolute risks determined and validated in two cohorts. It is built from reliable clinical variables that are usually examined among patients receiving PD care, implying that it can be readily applied in clinical practice and is amenable to further external validation in many regions and countries that provide routine PD care.

The methods used to derive and validate the model are similar to those for other risk prediction algorithms derived from the CPRD and QResearch databases^11,12^. The dataset used in this dataset was the largest dataset used for risk predictions among patients receiving PD care. The HPRD is the only PD registry data in Henan including all patients receiving PD care in Henan who will be followed up for their lifetime; therefore selection bias and respondent bias were relatively small in this study. The HPRD is located in Henan, the province with the largest population size in China, suggesting our study is likely to be a representativeness sample.

There were some limitations in our study. First, there was some distinctive difference between the patients in our study and typical European ESRD patients, for example, young age, lower BMI, lower prevalence of comorbidities and lower percentage of treatment, which suggested potential adjustments might be needed when applied our risk algorithm into external ESRD population, especially European ESRD populations. Second, some traditional risk factors, like smoking and prior health information were not accessible in our study. Third, the relative high missing percentage of some variables, for example, phosphorus and albumin might have some impact on extrapolation of our models especially in the external population, our risk algorithms were derived from imputed datasets though. Fourth, although our risk algorithm was helpful in populations where the access or the validity of previous information on cardiovascular risk factors and comorbidity were restricted, the external validation in more typical cohorts would still be warranted. Fifth, the threshold of absolute risk to define “high-risk” patients was not provided in this study, several thresholds as illustrations were provided though, as the definition would need to balance risks and benefits for patients and analyse cost effectiveness, which exceeded our study scope. Sixth, there were some calibration differences observed between the derivation and validation cohorts, with some predictors, particularly, higher transferrin levels, observed in the validation cohort.

### The selection of predictors

All available variables in HPDR were reviewed by 5 independent clinicians and processed as candidate predictors following a consensus process. Our predictive model differs from existing algorithms developed in developed countries, by excluding multimorbidities, to take account of the difficulty in accessing such data which are not routinely recorded in the under-developed primary care system in China. The comorbidities, like existing cardiovascular disease, were eliminated by the final model, which might be explained by they are both common between patients with and without outcomes^13^.

The majority of predictors in our final model incorporated accurate and reliable clinical measurements^9^, which were usually examined at the time of entry into PD and were more likely to be accessible across PD clinics in China^5^. Moreover, our limited utilisation of self-reported information should lead to minimal recall bias. Our predictive algorithm can be easily validated with other external datasets.

### Comparison with other studies

Mortality among patients undergoing PD varies with the ethnicity of the population, the characteristics within different datasets and lengths of follow-up time^14^. The PD registration dataset reported 29.7% all-cause mortality within 3 years^7^ and another Chinese study presented all-cause mortality as 19.4% within two years^8^. Cardiovascular mortality in a small dataset from Hong Kong, China, was 23.8% within 4 years^15^. On consideration of the lengths of follow-up time, our cardiovascular mortality of 14%, was comparable to other studies in Chinese PD populations.

In the risk prediction models for all-cause mortality in people receiving PD care, both the haemoglobin and albumin have been consistently associated with all-cause mortality^6,7,15^, as found in our study. In another Chinese cohort, fasting glucose, and diastolic blood pressure were also used in the prediction algorithm^8^, with similar associations identified in our study. Other established risk factors for cardiovascular mortality, like low density lipoprotein, systolic blood pressure, total cholesterol, and body mass index in general population were also utilised in our predictive algorithms^16,17^. Some novel predictors were introduced in our model, for example, total protein, sodium, phosphate. Both creatinine and eGFR used in prediction of all-cause mortality^18,19^ were eliminated by our final model. Different outcomes and variation between of datasets could be the reason^20^, and the sole utilisation of complete datasets or drop-off participants with missing values could be another reason^21^. In our prediction model, according to TRIPOD guidance^14^, we reported the percentage of missing variables and implemented multiple imputation both in our derivation and validations cohorts. In the model with imputed datasets, both creatinine and eGFR were eliminated by the final model. Unlike prediction tools developed in developed countries, little information on comorbidities or lifestyle factors were utilised in our model (like other models derived in Chinese populations) due to the difference in the care system and inaccurate self-reported information^9,22^.

## Conclusion

We have developed and validated new risk prediction equations to quantify the absolute risks of cardiovascular mortality within two years in patients initialising PD care. Our study has two important implications for clinical practices. Firstly, our prediction model can be used as a tool to identify patients at high risk of cardiovascular mortality within two years of initiation of PD care. The algorithms are based on standard clinical measurements that are likely to be available at the point of initialisation of PD care. Secondly, our risk prediction model could be used to establish new treatment thresholds in clinical practice through consensus development of national guidance to provide intensive cardio-protective intervention to improve the survival probability in patients with high risks of cardiovascular mortality.

## Methods

### Data source and study population

For this study, we used data from the Henan Peritoneal Dialysis Registry (HPDR) to develop and validate the risk score. Henan is a province in the Central of China with the population over 100 million. Briefly, the HPDR is operated under the auspices of the Department of Nephrology, the First Affiliated Hospital of Zhengzhou University and provides an independent audit and analysis of renal care in Henan, China. During the study period, information was prospectively collected electronically from all renal units across Henan. Data arriving at the HPDR are subjected to an algorithm which identifies suspicious values, which are then further verified and corrected where necessary by contacting the renal unit. This study was designed as a cohort study, which included all adults aged more than 18 years who commenced PD between 2007–2014 and who had at least two years’ follow-up. Patients who died, underwent transplant or whose kidney function recovered within 90 days after initialisation of dialysis were excluded (n = 16) *to avoid a reverse causality association between predictors and outcome*. This reflects the standard approach to investigating “real” ESRD patients among all those receiving PD care. We randomly allocated two thirds of patients to the derivation dataset and the remaining one third to a validation dataset. Ethics approval was granted by the Clinical Research Ethics Committee of the First Affiliated Hospital of Zhengzhou University. Written informed consent was obtained from all participants before inclusion.

### Defining outcome, predictors, missing data and power calculation

We defined our primary outcome as recorded death with clinically diagnosed cardiovascular disease^23,24^. All available information, including demographic characteristics, self-reported comorbidities, and clinical measurements at the time of patients initialising the PD were evaluated by 5 clinicians. Predictors with agreement (≥3 clinicians) were included in the analysis for further evaluation as candidate predictors. Backward elimination in a multivariate logistic regression model, with inclusion of all candidate predictors, was applied to select the predictors for the final model. For predictors used in the final model, our derivation cohort had missing information on body mass index (13.51%), phosphorus (20.92%), albumin (19.92%), total protein (22.57%), total cholesterol (24.25%), low density lipoprotein (24.58%), fasting glucose (15.92%), sodium (8.02%), systolic blood pressure (4.82%), and diastolic blood pressure (4.82%). We used multiple imputation to replace missing values by using a chained equation approach based on all candidate predictors. We created 30 imputed datasets for missing variables that were then combined across all datasets by using Rubin’s rule to obtain final model estimates. With 261 cardiovascular deaths during the first two years of initiation of PD and 12 predictors in the final derivation cohort, we had an effective sample size of 14 final events per predictor, above the minimum requirement suggested by Peduzzi *et al*.^25^.

### Statistical analysis for model derivation and validation

The methodology used in a previous prediction model was used in this study to derive and validate our risk algorithm^12^. We treated CVD mortality during the first two years of initialisation of PD as a binary outcome measure. For each candidate variable, we used a univariable logistic regression model to calculate the unadjusted odds ratio. Through backward elimination, we excluded candidate predictors from the multivariable model that were not statistically significant (P > 0.1 based on change in log likelihood)^26^. After elimination, we reinserted excluded predictors into the final model to further check whether they became statistically significant. We used fractional polynomials to model potential non-linear relations between outcome and continuous variables^11^. We also re-checked fractional polynomial terms at this stage and re-estimated them where necessary. We formed the risk equation for predicting the log odds of cardiovascular mortality by using the estimated β coefficients multiplied by the corresponding predictors included in our model, together with the average intercept across patient clusters. This process ultimately led to an equation for the predicted absolute risk of cardiovascular mortality: predicted risk = 1/(1 + e-^riskscore^), where the “risk score” is the predicted log odds of cardiovascular mortality from the developed model^12^.

We assessed the performance of the model in terms of the C statistic and calibration slope. The C statistic represents the probability that for any randomly selected patient with or without final event, the patient who had a final event had a higher predicted risk. A value of 0.50 represents no discrimination and 1.00 represents perfect discrimination. We then did internal validation to correct measures of predictive performance for optimism (over-fitting) by bootstrapping 100 samples of the derivation data. We repeated the model development process in each bootstrap sample (as outlined above, including variable selection) to produce a model, applied the model to the same bootstrap sample to quantify apparent performance, and applied the model to the original dataset to test model performance (calibration slope and C statistic) and optimism (difference in test performance and apparent performance). We then estimated the overall optimism across all models (for example, derive shrinkage coefficient = average calibration slope from each of the bootstrap samples)^14^. To account for over-fitting during the development process, we multiplied the original β coefficient by the uniform shrinkage factor in the final model. We re-estimated the intercept on the basis of the shrunken β coefficients to ensure that overall calibration was maintained, producing a final model.

We applied our risk prediction model to each patient in the validation cohort on the basis of the presence of one or more risk factors (Box 1). We examined the performance of this final model in terms of discrimination by calculating C statistics. We examined calibration by plotting agreement between predicted and observed risks across tenth of predicted risk.

We used Stata version 14 for all statistical analyses. This study was conducted and reported in line with the Transparent Reporting of a multivariate prediction model for individual Prediction or Diagnosis (TRIPOD) guidelines^27^.

### Data Availability

The datasets generated during and/or analysed during the current study are available from the corresponding author on reasonable request.

## Electronic supplementary material

Supplementary information
