# Author Correction: Development and validation of risk prediction models for cardiovascular mortality in Chinese people initialising peritoneal dialysis: a cohort study

**DOI:** 10.1038/s41598-018-23239-z

**Published:** 2018-04-12

**Authors:** Dahai Yu, Yamei Cai, Ying Chen, Tao Chen, Rui Qin, Zhanzheng Zhao, David Simmons

**Affiliations:** 1 Department of Nephrology, the First Affiliated Hospital, Zhengzhou University, Zhengzhou, 450052 China; 2 Arthritis Research UK Primary Care Centre, Research Institute for Primary Care & Health Sciences, Keele University, Keele, ST5 5BG UK; 3 Tropical Clinical Trials Unit, Department of Clinical Sciences, Liverpool School of Tropical Medicine, Pembroke Place, Liverpool, L3 5QA UK; 4 Jiangsu Province Hospital on Integration of Chinese and Western Medicine, Nanjing, 210028 China; 5 Western Sydney University, Campbelltown, Sydney, NSW 2751 Australia

Correction to: *Scientific Reports* 10.1038/s41598-018-20160-3, published online 31 January 2018

The original version of this Article contained an error in the order of author names, which were incorrectly given as ‘Dahai Yu, Yamei Cai, Ying Chen, Tao Chen, Rui Qin, David Simmons & Zhanzheng Zhao’.

Additionally, this Article contained a typographical error in Table 3.

‘validatio’

now reads:

‘Validation’

These errors have now been corrected in the HTML and PDF versions of this Article, and in the accompanying Supplementary Information file.

