# Supplementary material for: Development and validation of risk prediction models for cardiovascular mortality in Chinese people initialising peritoneal dialysis: a cohort study
Source: Sci Rep. 2018 Jan 31;8:1966. doi: 10.1038/s41598-018-20160-3 (PMC5792639; doi:10.1038/s41598-018-20160-3)
Supplement: Supplementary file 1 — Supplementary information [file 41598_2018_20160_MOESM1_ESM.pdf]

Title: Development and validation of risk prediction models for cardiovascular mortality in Chinese people initialising peritoneal dialysis: a cohort study

Authors: Dahai Yu, PhD [1,2], Yamei Cai, MD [1], Ying Chen, PhD [1,2], Tao Chen, PhD [1,3] , Rui Qin, MD [4], Zhanzheng Zhao, PhD [1*], David Simmons, MD (Cantab) [5*],

1. Department of Nephrology, the First Affiliated Hospital, Zhengzhou University, Zhengzhou 450052, China

2. Arthritis Research UK Primary Care Centre, Research Institute for Primary Care & Health Sciences, Keele University, Keele ST5 5BG, UK

3. Tropical Clinical Trials Unit, Department of Clinical Sciences, Liverpool School of Tropical Medicine, Pembroke Place, Liverpool L3 5QA, UK

4. Jiangsu Province Hospital on Integration of Chinese and Western Medicine, Nanjing 210028, China

5. Western Sydney University, Campbelltown, Sydney NSW 2751, Australia

***Correspondence (China):**
Professor Zhanzheng Zhao, Department of Nephrology, the First Affiliated Hospital Zhengzhou University, Zhengzhou 450052, CHINA
Email: zhanzhengzhao@zzu.edu.cn
TEL:+86 139 3852 5666
FAX:+86 371 6698 8753

***Correspondence (Australia):**
Professor David Simmons, Macarthur Clinical School, School of Medicine, Western Sydney University, Locked Bag 1797, Campbelltown NSW 2751, AUSTRALIA
Email: dsworkster@gmail.com
TEL: (61+2) 4620 3899
FAX: (61+2) 4620 3890

Words count: Abstract: 222 Main text 2,333

References 27 Tables 4 Figures 1 Online-supplemental: 1

Running headline: Predicting CV deaths in patients starting PD care

Online supplemental

Table-S1. Unadjusted odds ratio for cardiovascular mortality within 2 years of initialisation of peritoneal dialysis in the derivation cohort

Predictor	Coefficient	95% Confidence Interval
Age	0.024822	(0.023863 to 0.02578)
sex	-0.274890	(-0.30309 to -0.24669)
Haemoglobin, g/L	-0.002730	(-0.00342 to -0.00204)
Packed cell volume	-0.010840	(-0.01201 to -0.00967)
Reticulocyte, %	0.010404	(0.009705 to 0.011104)
Phosphorus, mg/dl	0.169103	(0.149101 to 0.189104
Alumbin, g/L	-0.071020	(-0.07319 to -0.06885)
Total iron binding capacity , µmol/L	0.010848	(0.010079 to 0.011617)
FeTIBC, mmol/L	-0.013470	(-0.01503 to -0.0119)
Creatinine, mmol/L	0.000150	(0.000132 to 0.000167)
estimated Glomerular Filtration rate, mL/min/1.73 m^2	0.006038	(0.005344 to 0.006731)
Transferrin, mg/dl	0.000352	(0.000294 to 0.00041)
Total protein, g/L	-0.018220	(-0.01972 to -0.01672)
Prealbumin, mg/L	-0.000920	(-0.00109 to -0.00076)
Total Cholesterol, mmol//L	-0.059880	(-0.07022 to -0.04954)
Low density lipoprotein, mmol//L	-0.068850	(-0.07811 to -0.05959)
Fasting glucose, mmol/L	0.052735	(0.048058 to 0.057412)
Sodium, mEq/L	-0.032180	(-0.0345 to -0.02987)
C-reaction protein, mg/dl	0.000141	(-0.00089 to 0.001176)
Body mass index, kg/m^2	-0.008770	(-0.01304 to -0.00451)
Systolic blood pressure, mmHg	-0.001030	(-0.00175 to -0.00031)
Diastolic blood pressure, mmHg	-0.009570	(-0.01065 to -0.00849)
Cardiovascular disease, yes vs no	0.237291	(0.200599 to 0.273983)
Diabetes mellitus, yes vs no	0.443774	(0.397027 to 0.49052)
Primary Glomerular Disease, yes vs no	-0.427822	(-0.466305 to -0.389339)
Taking antihypertensive treatment, yes vs no	0.207039	(-0.17025122 to 0.58432903)

Box-S1. Risk prediction model

Risk score from a logistic regression model to predict cardiovascular mortality in the first two years of initialisation of peritoneal dialysis. Risk score=4.08926-0.5102×((Body mass index/10)^2)+ 0.35498×((Body mass index/10)^2×ln(Body mass index/10)) -0.582×((Phosphorus/10)^-0.5)+ 2.60171×((Phosphorus/10)^3) -0.002×((Total Protein/100)^-2) -0.6709×((Total Protein/100)^-0.5)+ 0.91848×([(Total Cholesterol+1.673428429186277)/10]^0.5) -1.3419×([(Total Cholesterol+1.673428429186277)/10]^0.5*ln[(Total Cholesterol+1.673428429186277)/10]) - 1.0792×((Sodium/100)^3)+ 1.09368×((Sodium/100)^3×ln(Sodium/100)) -0.2414×((Systolic blood pressure/100)^-2) -0.6225×((Systolic blood pressure/100)^-2×ln(Systolic blood pressure/100)) - 0.4171×((Diastolic blood pressure/100)^-2) -0.4521×((Diastolic blood pressure/100)^-2*ln(Diastolic blood pressure/100)) -0.0791×Albumin-0.0679×Low density lipoprotein+0.01807×Fastinig glucose

Categorical variables were coded as binary (0 or 1 for absence or presence of a risk factor). Body mass index, phosphorus, total protein, total cholesterol, sodium, systolic blood pressure and diastolic blood pressure were transformed on the basis of fractional polynomial regression analysis. The value 4.08926 is the intercept, and other numbers are the estimated regression coefficients for the predictors, which indicate their mutually adjusted relative contribution to the outcome risk. The regression coefficients represents the log odds ratio for a change of 1 unit in the corresponding predictor. The predicted risk of cardiovascular mortality =1/(1+e$^{-riskscore}$).

Example -A 40 year old male patient whose body mass index, phosphorus, total protein, total cholesterol, sodium, systolic blood pressure, diastolic blood pressure albumin, low density lipoprotein and fasting glucose was 23, 2, 60, 5, 142, 150mmHg, 100mmHg, 35, 3 mmol/L and 5.8 mmol/L. He had a predicted risk of 16.2% of developing to cardiovascular mortality in the first two years of initialisation of PD care. Interpretation: if 1000 patients with this same risk factors are followed, 162 will develop cardiovascular mortality with the first two years of initialisation of PD care.
